# Hemorrhage after Extraction

**Published:** 1899-04

**Authors:** 


ARTICLE II.

### HEMORRHAGE AFTER EXTRACTION.

It is a most fortunate matter for the dental profession that cases of hemorrhage after extraction are as a rule, very few and far between, but such cases occasionally occur, and it is well to be prepared to deal with them efficiently. Of course, as most dentists are aware, there are a class of patients who are called bleeders, who have what is termed a hemorrhage diathesis, a state of the blood which predisposes to severe and dangerous loss of blood, no matter in what part of the body the wound may be situated, and in some patients, who are not of this class the cause is the result of a want of coagulating power in the fibrine or molecules in the blood; it has been said a want of iron. I also find that as a rule the first and second lower molars are most frequently the teeth that cause most trouble in this respect, and, as in other diseases some families are more predisposed to hemorrhage than others. There are various remedies or styptics, as they are called, but I find the most successful, if properly applied, is perchloride of iron. It is well to see that the cavity is thoroughly cleaned out, all clots of blood removed, and a good solid plug of cotton-wool dipped in the styptic, pressed into the cavity. Unfortunately, patients frequently allow the bleeding to go on for several days before returning to have it stopped, and the longer it goes on the more difficult it is to remedy. Others, again, instead of returning to you, go to a doctor, and blame you for malpraxis.

A gentleman, about 55 years of age, robust, and with florid complexion, came to me and had four decayed teeth extracted, one of them being a lower molar. After they were extracted there did not appear to be any undue bleeding, and he left about three o'clock. About seven o'clock his wife called and told me he had been bleeding all the afternoon, and it seemed to get worse, which alarmed them. I at once took a few instruments, some cotten-wool, and perchloride of iron. When we reached the house, I found the blood still flowing freely. I cleaned out the cavity well, and filled with a good firm plug of cotton-wool soaked in the perchloride of iron. After seeing the bleeding was thoroughly stopped, I left. Between two and three o'clock next morning the bell rang; I rose, and went to the door to find my patient leaning over the gate in a fainting condition. I assisted him into the house, and gave him a little stimulant to rouse him. He then became sick, and vomited a large quantity of pure blood. I then proceeded to clean out the clots from the cavity, and re-plugged it again with cotton and perchloride of iron, over which I put a pad of linen cloth also soaked in the solution, made him close his teeth on it, and bandaged the jaws together, telling him to sit up in an easy-chair till morning. He told me it was all right till he got into bed, when the plug came out, and, so as not to disturb his wife, he kept swallowing the blood till at last he was compelled to rise and come to me. I called in the morning, and found there had been no recurrence of the hemorrhage.

Another case was that of a lady who called between nine and ten one night to have a lower molar extracted. In the middle of the night her servant came for me, and said her mistress was bleeding to death. I went with her and, as in the previous case, cleaned the clots well out, and firmly plugged the cavity, telling her on no account to go to bed, but sit up in a chair all night. I found she had lost a very large quantity of blood, and with every pulsation of the heart could see the blood welling up. I also, in such cases, recommend the patient to take internally tincture of iron for a time. From these and other similar cases, I have come to the conclusion that it is unwise to extract teeth at night when it is near bed-time, no matter though the patient may not be predisposed to bleeding, as in any case the recumbent position is dangerous. Where there is an open wound in the mouth it is safest to fill the cavity in the tooth with a palliative such as cotton-wool dipped in sandarac varnish till next forenoon, when it can be extracted safely; besides, it is a difficult matter extracting teeth at night, as the mouth is a dark place at any time. Another thing, I have found that those who smoke are apt to be troubled with persistent hemorrhage after extraction, from the constant suction.

The following case is rather remarkable, being one of vicarious hemorrhage after the extraction of a tooth. In the Deutsche Vierteljahrsschrift, Herr Rabud gives the following case: The patient was a young girl, 14 years of age. Eight days after the extraction of a tooth very profuse hemorrhage from the alveolus occurred, the menstrual discharge which had commenced the day before being thereby reduced to a minimum; on the hemorrhage in the mouth being arrested, the menstrual flow returned.—*Dens Sapientiae, in the Dentist.*

